# Retraction Notice to: Preparation, phase stability, and magnetization behavior of high entropy hexaferrites

**DOI:** 10.1016/j.isci.2026.115383

**Published:** 2026-03-19

**Authors:** Vladimir E. Zhivulin, Evgeniy A. Trofimov, Olga V. Zaitseva, Daria P. Sherstyuk, Natalya A. Cherkasova, Sergey V. Taskaev, Denis A. Vinnik, Yulia A. Alekhina, Nikolay S. Perov, Kadiyala C.B. Naidu, Halima I. Elsaeedy, Mayeen U. Khandaker, Daria I. Tishkevich, Tatiana I. Zubar, Alex V. Trukhanov, Sergei V. Trukhanov

## Main text

(iScience *26*, 107077; July 21, 2023)

This article has been retracted at the request of the editor. The decision to retract this article is due to issues that have arisen in relation to its content. After publication, concerns were raised regarding several figures and tables. Specifically, XRD patterns, unit cell parameter data, SEM images, and multiple figures and tables show strong similarities to those published in a previous article in *Ceramics International* (Zhivulin et al., 2023, Ceramics International *49*, 1069–1084, https://doi.org/10.1016/j.ceramint.2022.09.082), with only minor modifications such as changes in image color and decimal formatting and other similar alterations, but without proper citation or permission. The journal editor asked the authors to explain these concerns. Although a response was provided by the corresponding author, following a careful and thorough evaluation of the article, the editor lost confidence in the integrity of the article and decided to retract it. The authors did not respond regarding the decision to retract.

